# *rac*-(3a*R*,6a*R*)-(*E*)-Methyl 2-(3a-methyl­perhydro­furo[3,2-*b*]furan-2-yl­idene)acetate

**DOI:** 10.1107/S1600536810032101

**Published:** 2010-08-21

**Authors:** Lenka Bellovičová, Jozef Kožíšek, Jana Doháňošová, Angelika Lásiková, Tibor Gracza

**Affiliations:** a Department of Physical Chemistry, Faculty of Chemical and Food Technology, Slovak University of Technology, Radlinského 9, SK-812 37 Bratislava, Slovak Republic; b Department of Organic Chemistry, Faculty of Chemical and Food Technology, Slovak University of Technology, Radlinského 9, SK-812 37 Bratislava, Slovak Republic

## Abstract

The constitution and relative configuration at the stereogenic centres and stereochemistry of the C—C double bond formed during Pd^II^-catalysed domino reaction was established by X-ray analysis of the title compound, C_10_H_14_O_4_. The asymmetric unit contains two mol­ecules.

## Related literature

The title compound was prepared from 4-methyl­pent-4-en-1,3-diol (Breit & Zahn, 2001 ▶) by a modified procedure for carbonyl­ation of alkene-3-ol (Semmelhack & Epa, 1993 ▶).
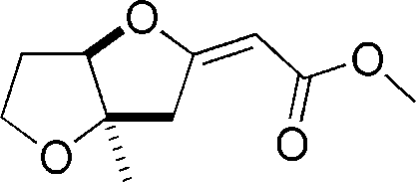


## Experimental

### 

#### Crystal data

C_10_H_14_O_4_
*M* *_r_* = 198.21
Monoclinic, 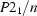

*a* = 12.159 (1) Å
*b* = 5.8100 (3) Å
*c* = 28.509 (1) Å
β = 101.51 (1)°
*V* = 1973.5 (2) Å^3^
*Z* = 8
Mo *K*α radiation
μ = 0.10 mm^−1^
*T* = 293 K
0.84 × 0.36 × 0.12 mm

#### Data collection

Oxford Diffraction Gemini R CCD diffractometer
Absorption correction: analytical [*CrysAlis PRO* (Oxford Diffraction, 2010 ▶); analytical numeric absorption correction using a multi-faceted crystal model based on expressions derived by Clark & Reid (1995 ▶)] *T*_min_ = 0.941, *T*_max_ = 0.988
59312 measured reflections
4033 independent reflections
3571 reflections with *I* > 2σ(*I*)
*R*_int_ = 0.024

#### Refinement

*R*[*F* ^2^ > 2σ(*F* ^2^)] = 0.036
*wR*(*F* ^2^) = 0.091
*S* = 1.05
4025 reflections
254 parameters
H-atom parameters constrained
Δρ_max_ = 0.32 e Å^−3^
Δρ_min_ = −0.22 e Å^−3^

### 

Data collection: *CrysAlis CCD* (Oxford Diffraction, 2009 ▶); cell refinement: *CrysAlis CCD*; data reduction: *CrysAlis RED* (Oxford Diffraction, 2009 ▶); program(s) used to solve structure: *SHELXS97* (Sheldrick, 2008 ▶); program(s) used to refine structure: *SHELXL97* (Sheldrick, 2008 ▶); molecular graphics: *DIAMOND* (Brandenburg, 1998 ▶); software used to prepare material for publication: *enCIFer* (Allen *et al.*, 2004 ▶).

## Supplementary Material

Crystal structure: contains datablocks global, I. DOI: 10.1107/S1600536810032101/bv2142sup1.cif

Structure factors: contains datablocks I. DOI: 10.1107/S1600536810032101/bv2142Isup2.hkl

Additional supplementary materials:  crystallographic information; 3D view; checkCIF report

## Figures and Tables

**Fig. 1.**
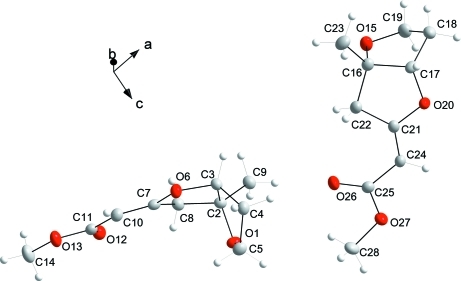
The numbering scheme of title compound. Displacement ellipsoids are shown at the 50% probability level.

**Fig. 2.**
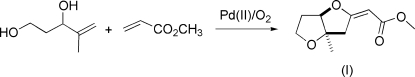
Synthesis of rac-(3aR, 6aR)-(E)-methyl 2-(3a-methyl-tetrahydrofuro [3,2-b]furan-2-ylidene)acetate.

## supplementary crystallographic information

### Comment

As a part of our long term program directed towards the application of palladium(II)-catalysed oxycarbonylation of unsaturated polyols in the natural product synthesis we studied the domino Pd(II)-promoted reactions. The title compound, [(I): alternative name: (±)-(1'*R*, 5'*R*)-(*E*)-methyl 2-(5'-methyl-2',6'-dioxabicyclo[3.3.0]octa-3'-ylidene) acetate] represents a product of the first diastereoselective domino intramolecular Wacker-type cyclization - Heck reaction - cyclization of 4-methylpent-4-en-1,3-diol with methyl acrylate. The asymmetric unit contains two molecules of the same chirality (*Z*' = 2), but as the space group is centrosymmetric, both enantiomers are present in the unit cell.

### Experimental

The title compound was prepared from 4-methylpent-4-en-1,3-diol (Breit and Zahn, 2001) by a modified procedure for carbonylation of alkene-3-ol (Semmelhack and Epa, 1993). A mixture of 4-methylpent-4-en-1,3-diol (200 mg, 1.70 mmol, 1 equivalent) and CuCl freshly recrystallized (170 mg, 1.70 mmol, 1 equivalent) in dry DMF (7 ml) was stirred at r.t for 10 min. under oxygen atmosphere (balloon). The methyl acrylate (0.8 ml, 8.60 mmol, 5 equivalents) and palladium acetate (39 mg, 0.17 mmol, 0.1 equivalent) were then added. The mixture was stirred for 56 h, then diluted by ethyl acetate (100 ml). The organic solution was washed three times with sat. aq. ammonium chloride solution, driedover anhydrous magnesium sulfate, and concentrated *in vacuo*. The residue was purified by flash chromatography (SiO_2_, ethyl acetate-hexane-3:1, Rf 0.73). The title compound was slowly crystallized from hexane to give white crystals [m.p. 65–67 °C].^1^H NMR (300 MHz, Varian, CDCl_3_): δ(p.p.m.) = 1.43 (s, 3H, CH_3_); 2.17 (m, 2H, H-8'); 2.96 3.64 (2xd, 2H, J=19.7, H-4'); 3.69–4.04 (m, 6H, H-1', H-7', OCH_3_); 4.66 (d, 1H, J= 6.3 Hz, H-2). ^13^C NMR (75 MHz, CDCl_3_): δ(p.p.m.) = 22.7 (q, CH_3_), 32.7 (t, C-8'), 44.2 (t, C-4'), 50.8 (q, OCH_3_), 66.9 (t, C-7'), 87.7 (s, C-5'), 89.9 (d, C-1'), 91.3 (d, C-2), 168.7 (s, C-1), 175.7 (d, C-3'). IČ, film: ν(cm^-1^) = 3479 (w), 2975 (*m*), 2951 (*m*), 2874 (w), 1789 (w), 1705 (*s*), 1645 (*s*), 1437 (*s*), 1410 (w), 1364 (*s*), 1317 (*m*), 1274 (*m*), 1193 (*s*), 1148 (*s*), 1106 (*s*), 1093 (*s*), 1039 (*s*), 1010 (*m*), 978 (*m*), 950 (w), 933 (w), 900 (w), 871 (w), 822 (*m*), 734 (w), 592 (w) [cm^-1^]

### Refinement

All H atoms were placed in geometrically idealized positions and constrained to ride on their parent atoms (C—H = 0.93, 0.96 and 0.97 Å) and *U*_iso_(H) values were taken to be equal to 1.2 *U*_eq_(C) all H atoms.

### Crystal data

**Table:**

C_10_H_14_O_4_	*F*(000) = 848
*M**_r_* = 198.21	*D*_x_ = 1.334 Mg m^−^^3^
Monoclinic, *P*2_1_/*n*	Mo *K*α radiation, λ = 0.71073 Å
Hall symbol: -P 2yn	Cell parameters from 35551 reflections
*a* = 12.159 (1) Å	θ = 3.6–29.5°
*b* = 5.8100 (3) Å	µ = 0.10 mm^−^^1^
*c* = 28.509 (1) Å	*T* = 293 K
β = 101.51 (1)°	Block, colorless
*V* = 1973.5 (2) Å^3^	0.84 × 0.36 × 0.12 mm
*Z* = 8	

### Data collection

**Table:**

Oxford Diffraction Gemini R CCD diffractometer	4033 independent reflections
Radiation source: Enhance (Mo) X-ray Source	3571 reflections with *I* > 2σ(*I*)
graphite	*R*_int_ = 0.024
Detector resolution: 10.4340 pixels mm^-1^	θ_max_ = 26.4°, θ_min_ = 3.6°
Rotation method data acquisition using ω and φ scans	*h* = −15→15
Absorption correction: analytical [*CrysAlis PRO* (Oxford Diffraction, 2010); analytical numeric absorption correction using a multi-faceted crystal model based on expressions derived by Clark & Reid (1995)]	*k* = −7→7
*T*_min_ = 0.941, *T*_max_ = 0.988	*l* = −35→35
59312 measured reflections	

### Refinement

**Table:**

Refinement on *F*^2^	Secondary atom site location: difference Fourier map
Least-squares matrix: full	Hydrogen site location: inferred from neighbouring sites
*R*[*F*^2^ > 2σ(*F*^2^)] = 0.036	H-atom parameters constrained
*wR*(*F*^2^) = 0.091	*w* = 1/[σ^2^(*F*_o_^2^) + (0.0375*P*)^2^ + 1.2579*P*] where *P* = (*F*_o_^2^ + 2*F*_c_^2^)/3
*S* = 1.05	(Δ/σ)_max_ = 0.001
4025 reflections	Δρ_max_ = 0.32 e Å^−^^3^
254 parameters	Δρ_min_ = −0.22 e Å^−^^3^
0 restraints	Extinction correction: *SHELXL97* (Sheldrick, 2008), Fc^*^=kFc[1+0.001xFc^2^λ^3^/sin(2θ)]^-1/4^
Primary atom site location: structure-invariant direct methods	Extinction coefficient: 0.0031 (7)

### Special details

**Table:**

Experimental. CrysAlisPro, Oxford Diffraction Ltd., Version 1.171.33.62 (release 16-03-2010 CrysAlis171 .NET) (compiled Mar 16 2010,16:26:05) Analytical numeric absorption correction using a multifaceted crystal model based on expressions derived by R.C. Clark & J.S. Reid. (Clark, R. C. & Reid, J. S. (1995). Acta Cryst. A51, 887-897)
Geometry. All e.s.d.'s (except the e.s.d. in the dihedral angle between two l.s. planes) are estimated using the full covariance matrix. The cell e.s.d.'s are taken into account individually in the estimation of e.s.d.'s in distances, angles and torsion angles; correlations between e.s.d.'s in cell parameters are only used when they are defined by crystal symmetry. An approximate (isotropic) treatment of cell e.s.d.'s is used for estimating e.s.d.'s involving l.s. planes.
Refinement. Refinement of *F*^2^ against ALL reflections. The weighted *R*-factor *wR* and goodness of fit *S* are based on *F*^2^, conventional *R*-factors *R* are based on *F*, with *F* set to zero for negative *F*^2^. The threshold expression of *F*^2^ > σ(*F*^2^) is used only for calculating *R*-factors(gt) *etc*. and is not relevant to the choice of reflections for refinement. *R*-factors based on *F*^2^ are statistically about twice as large as those based on *F*, and *R*- factors based on ALL data will be even larger.independent reflections were 4033, 7 inconsistent equivalents, 4025 were used in the refinement

### Fractional atomic coordinates and isotropic or equivalent isotropic displacement parameters (Å^2^)

**Table:**

	*x*	*y*	*z*	*U*_iso_*/*U*_eq_	
C2	0.35925 (10)	0.2368 (2)	0.34753 (4)	0.0173 (3)	
C3	0.35197 (10)	0.4998 (2)	0.34359 (4)	0.0174 (3)	
H3A	0.4074	0.5638	0.3265	0.021*	
C4	0.36899 (10)	0.5847 (2)	0.39470 (4)	0.0195 (3)	
H4B	0.3319	0.7311	0.3967	0.023*	
H4A	0.4481	0.5997	0.4089	0.023*	
C5	0.31418 (11)	0.3932 (2)	0.41825 (4)	0.0212 (3)	
H5B	0.3475	0.3829	0.4521	0.025*	
H5A	0.2344	0.4214	0.4149	0.025*	
C7	0.18323 (10)	0.3541 (2)	0.30141 (4)	0.0177 (3)	
C8	0.26323 (10)	0.1555 (2)	0.30792 (4)	0.0192 (3)	
H8B	0.2897	0.1237	0.2786	0.023*	
H8A	0.2282	0.0180	0.3175	0.023*	
C9	0.47359 (10)	0.1376 (2)	0.34558 (5)	0.0212 (3)	
H9C	0.4713	−0.0270	0.3484	0.025*	
H9B	0.5286	0.1989	0.3715	0.025*	
H9A	0.4933	0.1779	0.3157	0.025*	
C10	0.07393 (10)	0.3673 (2)	0.28094 (4)	0.0197 (3)	
H10A	0.0379	0.5089	0.2799	0.024*	
C11	0.01014 (10)	0.1674 (2)	0.26033 (4)	0.0189 (3)	
C14	−0.16945 (12)	0.0392 (3)	0.22261 (6)	0.0306 (3)	
H14C	−0.2446	0.0946	0.2118	0.037*	
H14B	−0.1693	−0.0791	0.2461	0.037*	
H14A	−0.1416	−0.0225	0.1960	0.037*	
C16	0.86595 (10)	0.9608 (2)	0.38508 (4)	0.0190 (3)	
C17	0.96259 (11)	1.0068 (2)	0.42723 (4)	0.0200 (3)	
H17A	0.9589	1.1601	0.4412	0.024*	
C18	1.06680 (11)	0.9721 (2)	0.40691 (5)	0.0247 (3)	
H18B	1.1303	0.9277	0.4316	0.030*	
H18A	1.0857	1.1098	0.3910	0.030*	
C19	1.03089 (11)	0.7781 (3)	0.37159 (5)	0.0255 (3)	
H19B	1.0714	0.7855	0.3456	0.031*	
H19A	1.0452	0.6299	0.3873	0.031*	
C21	0.85362 (10)	0.7177 (2)	0.45081 (4)	0.0177 (3)	
C22	0.78158 (10)	0.8268 (2)	0.40773 (4)	0.0206 (3)	
H22B	0.7264	0.9290	0.4169	0.025*	
H22A	0.7431	0.7111	0.3859	0.025*	
C23	0.81782 (12)	1.1718 (2)	0.35705 (5)	0.0270 (3)	
H23C	0.7581	1.1263	0.3314	0.032*	
H23B	0.8756	1.2467	0.3442	0.032*	
H23A	0.7893	1.2760	0.3779	0.032*	
C24	0.83161 (10)	0.5412 (2)	0.47796 (4)	0.0190 (3)	
H24A	0.8872	0.4903	0.5031	0.023*	
C25	0.72309 (11)	0.4285 (2)	0.46889 (4)	0.0201 (3)	
C28	0.61577 (12)	0.1322 (3)	0.49483 (5)	0.0279 (3)	
H28C	0.6234	0.0062	0.5170	0.033*	
H28B	0.5913	0.0751	0.4628	0.033*	
H28A	0.5615	0.2395	0.5021	0.033*	
O1	0.33343 (8)	0.18484 (15)	0.39400 (3)	0.0210 (2)	
O6	0.23724 (7)	0.54910 (15)	0.31945 (3)	0.0197 (2)	
O12	0.04472 (8)	−0.02687 (16)	0.25726 (3)	0.0231 (2)	
O13	−0.09856 (7)	0.22621 (17)	0.24354 (3)	0.0248 (2)	
O15	0.91270 (7)	0.81006 (17)	0.35392 (3)	0.0232 (2)	
O20	0.95504 (7)	0.82156 (16)	0.46110 (3)	0.0205 (2)	
O26	0.64051 (8)	0.48260 (19)	0.43955 (4)	0.0318 (3)	
O27	0.72256 (7)	0.24603 (16)	0.49859 (3)	0.0239 (2)	

### Atomic displacement parameters (Å^2^)

**Table:**

	*U*^11^	*U*^22^	*U*^33^	*U*^12^	*U*^13^	*U*^23^
C2	0.0185 (6)	0.0160 (6)	0.0180 (6)	−0.0016 (5)	0.0051 (5)	−0.0007 (5)
C3	0.0155 (6)	0.0168 (6)	0.0198 (6)	−0.0014 (5)	0.0032 (4)	0.0003 (5)
C4	0.0191 (6)	0.0178 (6)	0.0204 (6)	0.0004 (5)	0.0012 (5)	−0.0026 (5)
C5	0.0257 (6)	0.0192 (6)	0.0193 (6)	0.0023 (5)	0.0059 (5)	−0.0015 (5)
C7	0.0227 (6)	0.0163 (6)	0.0147 (5)	−0.0028 (5)	0.0050 (5)	−0.0003 (5)
C8	0.0192 (6)	0.0168 (6)	0.0217 (6)	−0.0007 (5)	0.0041 (5)	−0.0019 (5)
C9	0.0190 (6)	0.0199 (6)	0.0246 (6)	0.0001 (5)	0.0044 (5)	−0.0008 (5)
C10	0.0214 (6)	0.0181 (6)	0.0189 (6)	0.0016 (5)	0.0024 (5)	−0.0004 (5)
C11	0.0195 (6)	0.0226 (6)	0.0150 (5)	−0.0006 (5)	0.0041 (5)	0.0002 (5)
C14	0.0212 (7)	0.0321 (8)	0.0356 (8)	−0.0056 (6)	−0.0011 (6)	−0.0088 (6)
C16	0.0199 (6)	0.0196 (6)	0.0176 (6)	0.0025 (5)	0.0041 (5)	−0.0012 (5)
C17	0.0246 (6)	0.0179 (6)	0.0168 (6)	−0.0026 (5)	0.0022 (5)	0.0013 (5)
C18	0.0198 (6)	0.0295 (7)	0.0242 (6)	−0.0041 (5)	0.0029 (5)	0.0051 (6)
C19	0.0200 (6)	0.0307 (7)	0.0265 (7)	0.0047 (5)	0.0062 (5)	0.0011 (6)
C21	0.0163 (6)	0.0210 (6)	0.0160 (6)	0.0008 (5)	0.0032 (4)	−0.0031 (5)
C22	0.0178 (6)	0.0242 (6)	0.0195 (6)	0.0029 (5)	0.0029 (5)	0.0025 (5)
C23	0.0314 (7)	0.0261 (7)	0.0234 (6)	0.0069 (6)	0.0052 (5)	0.0065 (6)
C24	0.0166 (6)	0.0231 (6)	0.0165 (6)	0.0007 (5)	0.0012 (4)	0.0012 (5)
C25	0.0206 (6)	0.0233 (6)	0.0171 (6)	−0.0001 (5)	0.0055 (5)	−0.0002 (5)
C28	0.0256 (7)	0.0306 (7)	0.0281 (7)	−0.0105 (6)	0.0070 (5)	0.0010 (6)
O1	0.0282 (5)	0.0169 (4)	0.0198 (4)	−0.0007 (4)	0.0090 (4)	0.0002 (3)
O6	0.0196 (4)	0.0157 (4)	0.0214 (4)	−0.0003 (3)	−0.0013 (3)	−0.0007 (3)
O12	0.0236 (5)	0.0209 (5)	0.0239 (5)	−0.0012 (4)	0.0029 (4)	−0.0036 (4)
O13	0.0181 (4)	0.0262 (5)	0.0274 (5)	−0.0016 (4)	−0.0014 (4)	−0.0060 (4)
O15	0.0203 (5)	0.0283 (5)	0.0209 (4)	0.0026 (4)	0.0038 (4)	−0.0062 (4)
O20	0.0195 (4)	0.0232 (5)	0.0175 (4)	−0.0042 (4)	0.0003 (3)	0.0037 (4)
O26	0.0184 (5)	0.0438 (6)	0.0305 (5)	−0.0051 (4)	−0.0022 (4)	0.0121 (5)
O27	0.0211 (5)	0.0237 (5)	0.0264 (5)	−0.0046 (4)	0.0033 (4)	0.0048 (4)

### Geometric parameters (Å, °)

**Table:**

C2—O1	1.4529 (14)	C16—O15	1.4424 (15)
C2—C9	1.5164 (17)	C16—C23	1.5157 (18)
C2—C8	1.5283 (17)	C16—C17	1.5274 (17)
C2—C3	1.5333 (17)	C16—C22	1.5297 (17)
C3—O6	1.4555 (14)	C17—O20	1.4610 (15)
C3—C4	1.5131 (17)	C17—C18	1.5082 (18)
C3—H3A	0.9800	C17—H17A	0.9800
C4—C5	1.5203 (18)	C18—C19	1.517 (2)
C4—H4B	0.9700	C18—H18B	0.9700
C4—H4A	0.9700	C18—H18A	0.9700
C5—O1	1.4359 (15)	C19—O15	1.4369 (15)
C5—H5B	0.9700	C19—H19B	0.9700
C5—H5A	0.9700	C19—H19A	0.9700
C7—C10	1.3428 (18)	C21—C24	1.3433 (18)
C7—O6	1.3581 (15)	C21—O20	1.3516 (15)
C7—C8	1.4967 (17)	C21—C22	1.4994 (17)
C8—H8B	0.9700	C22—H22B	0.9700
C8—H8A	0.9700	C22—H22A	0.9700
C9—H9C	0.9600	C23—H23C	0.9600
C9—H9B	0.9600	C23—H23B	0.9600
C9—H9A	0.9600	C23—H23A	0.9600
C10—C11	1.4537 (17)	C24—C25	1.4494 (17)
C10—H10A	0.9300	C24—H24A	0.9300
C11—O12	1.2139 (16)	C25—O26	1.2131 (16)
C11—O13	1.3565 (15)	C25—O27	1.3576 (16)
C14—O13	1.4403 (16)	C28—O27	1.4420 (15)
C14—H14C	0.9600	C28—H28C	0.9600
C14—H14B	0.9600	C28—H28B	0.9600
C14—H14A	0.9600	C28—H28A	0.9600
			
O1—C2—C9	108.71 (10)	O15—C16—C22	109.33 (10)
O1—C2—C8	109.72 (10)	C23—C16—C22	114.41 (11)
C9—C2—C8	115.22 (10)	C17—C16—C22	103.44 (10)
O1—C2—C3	104.73 (10)	O20—C17—C18	108.89 (10)
C9—C2—C3	114.60 (10)	O20—C17—C16	104.54 (10)
C8—C2—C3	103.27 (10)	C18—C17—C16	104.34 (10)
O6—C3—C4	108.98 (10)	O20—C17—H17A	112.8
O6—C3—C2	105.46 (9)	C18—C17—H17A	112.8
C4—C3—C2	105.03 (10)	C16—C17—H17A	112.8
O6—C3—H3A	112.3	C17—C18—C19	101.64 (10)
C4—C3—H3A	112.3	C17—C18—H18B	111.4
C2—C3—H3A	112.3	C19—C18—H18B	111.4
C3—C4—C5	101.48 (10)	C17—C18—H18A	111.4
C3—C4—H4B	111.5	C19—C18—H18A	111.4
C5—C4—H4B	111.5	H18B—C18—H18A	109.3
C3—C4—H4A	111.5	O15—C19—C18	105.78 (11)
C5—C4—H4A	111.5	O15—C19—H19B	110.6
H4B—C4—H4A	109.3	C18—C19—H19B	110.6
O1—C5—C4	106.05 (10)	O15—C19—H19A	110.6
O1—C5—H5B	110.5	C18—C19—H19A	110.6
C4—C5—H5B	110.5	H19B—C19—H19A	108.7
O1—C5—H5A	110.5	C24—C21—O20	119.59 (11)
C4—C5—H5A	110.5	C24—C21—C22	130.07 (11)
H5B—C5—H5A	108.7	O20—C21—C22	110.34 (11)
C10—C7—O6	118.61 (11)	C21—C22—C16	103.27 (10)
C10—C7—C8	131.31 (12)	C21—C22—H22B	111.1
O6—C7—C8	110.06 (10)	C16—C22—H22B	111.1
C7—C8—C2	103.59 (10)	C21—C22—H22A	111.1
C7—C8—H8B	111.0	C16—C22—H22A	111.1
C2—C8—H8B	111.0	H22B—C22—H22A	109.1
C7—C8—H8A	111.0	C16—C23—H23C	109.5
C2—C8—H8A	111.0	C16—C23—H23B	109.5
H8B—C8—H8A	109.0	H23C—C23—H23B	109.5
C2—C9—H9C	109.5	C16—C23—H23A	109.5
C2—C9—H9B	109.5	H23C—C23—H23A	109.5
H9C—C9—H9B	109.5	H23B—C23—H23A	109.5
C2—C9—H9A	109.5	C21—C24—C25	121.40 (11)
H9C—C9—H9A	109.5	C21—C24—H24A	119.3
H9B—C9—H9A	109.5	C25—C24—H24A	119.3
C7—C10—C11	122.15 (12)	O26—C25—O27	121.75 (12)
C7—C10—H10A	118.9	O26—C25—C24	127.21 (12)
C11—C10—H10A	118.9	O27—C25—C24	111.04 (11)
O12—C11—O13	122.40 (12)	O27—C28—H28C	109.5
O12—C11—C10	127.40 (12)	O27—C28—H28B	109.5
O13—C11—C10	110.20 (11)	H28C—C28—H28B	109.5
O13—C14—H14C	109.5	O27—C28—H28A	109.5
O13—C14—H14B	109.5	H28C—C28—H28A	109.5
H14C—C14—H14B	109.5	H28B—C28—H28A	109.5
O13—C14—H14A	109.5	C5—O1—C2	110.45 (9)
H14C—C14—H14A	109.5	C7—O6—C3	111.12 (9)
H14B—C14—H14A	109.5	C11—O13—C14	114.63 (11)
O15—C16—C23	108.93 (10)	C19—O15—C16	110.61 (9)
O15—C16—C17	104.78 (10)	C21—O20—C17	111.13 (9)
C23—C16—C17	115.38 (11)	C25—O27—C28	115.34 (10)
			
O1—C2—C3—O6	−92.66 (10)	O20—C21—C22—C16	16.47 (13)
C9—C2—C3—O6	148.3 (1)	O15—C16—C22—C21	85.89 (11)
C8—C2—C3—O6	22.21 (12)	C23—C16—C22—C21	−151.64 (11)
O1—C2—C3—C4	22.4 (1)	C17—C16—C22—C21	−25.31 (12)
C9—C2—C3—C4	−96.62 (12)	O20—C21—C24—C25	178.52 (11)
C8—C2—C3—C4	137.27 (10)	C22—C21—C24—C25	−2.3 (2)
O6—C3—C4—C5	79.11 (11)	C21—C24—C25—O26	−4.7 (2)
C2—C3—C4—C5	−33.49 (12)	C21—C24—C25—O27	175.91 (11)
C3—C4—C5—O1	33.07 (12)	C4—C5—O1—C2	−20.25 (13)
C10—C7—C8—C2	−161.49 (13)	C9—C2—O1—C5	121.59 (11)
O6—C7—C8—C2	19.87 (13)	C8—C2—O1—C5	−111.59 (11)
O1—C2—C8—C7	86.28 (11)	C3—C2—O1—C5	−1.33 (13)
C9—C2—C8—C7	−150.66 (10)	C10—C7—O6—C3	175.36 (11)
C3—C2—C8—C7	−24.94 (12)	C8—C7—O6—C3	−5.81 (13)
O6—C7—C10—C11	179.36 (11)	C4—C3—O6—C7	−123.13 (11)
C8—C7—C10—C11	0.8 (2)	C2—C3—O6—C7	−10.82 (13)
C7—C10—C11—O12	−2.6 (2)	O12—C11—O13—C14	0.98 (17)
C7—C10—C11—O13	177.98 (11)	C10—C11—O13—C14	−179.57 (11)
O15—C16—C17—O20	−88.79 (11)	C18—C19—O15—C16	−17.45 (14)
C23—C16—C17—O20	151.4 (1)	C23—C16—O15—C19	119.00 (12)
C22—C16—C17—O20	25.73 (12)	C17—C16—O15—C19	−4.99 (13)
O15—C16—C17—C18	25.5 (1)	C22—C16—O15—C19	−115.31 (11)
C23—C16—C17—C18	−94.26 (13)	C24—C21—O20—C17	179.38 (11)
C22—C16—C17—C18	140.03 (11)	C22—C21—O20—C17	0.07 (14)
O20—C17—C18—C19	76.14 (12)	C18—C17—O20—C21	−127.75 (11)
C16—C17—C18—C19	−35.04 (13)	C16—C17—O20—C21	−16.70 (13)
C17—C18—C19—O15	32.43 (13)	O26—C25—O27—C28	−3.84 (18)
C24—C21—C22—C16	−162.75 (13)	C24—C25—O27—C28	175.63 (11)
